# Rapid intensification and the bimodal distribution of tropical cyclone intensity

**DOI:** 10.1038/ncomms10625

**Published:** 2016-02-03

**Authors:** Chia-Ying Lee, Michael K. Tippett, Adam H. Sobel, Suzana J. Camargo

**Affiliations:** 1 International Research Institute of Climate and Society, Columbia University, Palisades, New York 10964, USA; 2 Department of Applied Physics and Applied Mathematics, Columbia University, New York 10027, USA; 3 Center of Excellence for Climate Change Research, Department of Meteorology, King Abdulaziz University, Jeddah 21589, Saudi Arabia; 4 Division of Ocean and Climate Physics, Lamont-Doherty Earth Observatory, Columbia University, Palisades, New York 10964, USA

## Abstract

The severity of a tropical cyclone (TC) is often summarized by its lifetime maximum intensity (LMI), and the climatological LMI distribution is a fundamental feature of the climate system. The distinctive bimodality of the LMI distribution means that major storms (LMI >96 kt) are not very rare compared with less intense storms. Rapid intensification (RI) is the dramatic strengthening of a TC in a short time, and is notoriously difficult to forecast or simulate. Here we show that the bimodality of the LMI distribution reflects two types of storms: those that undergo RI during their lifetime (RI storms) and those that do not (non-RI storms). The vast majority (79%) of major storms are RI storms. Few non-RI storms (6%) become major storms. While the importance of RI has been recognized in weather forecasting, our results demonstrate that RI also plays a crucial role in the TC climatology.

## Abstract (web summary)

Tropical cyclones rarely achieve high intensities gradually. Here, the authors show that rapid intensification is relevant not only to short-term weather forecasting, but also to the relationship between tropical cyclones and climate.

## Graphical abstract

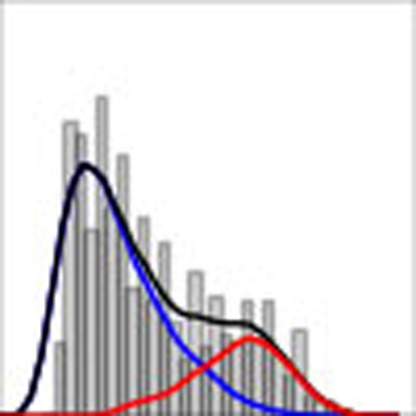

The question of how climate change will affect tropical cyclone (TC) activity has drawn considerable attention in the past two decades^1^,^2^,^3^,^4^. The current expectation is that we can expect a small increase in the global frequency of intense storms along with a small reduction in the total number of storms^4^. However, details of how the TC intensity distribution may change remain uncertain. This uncertainty reflects both the difficulty of simulating the most intense storms in climate change projections^4^, as well as an incomplete understanding of what determines the climatological intensity distribution in the current climate^5^. Improved understanding of the TC intensity distribution in the current climate seems necessary if we are to understand TC intensity changes in a warming climate. Among various metrics of TC intensity, lifetime maximum intensity (LMI) is an integrated statistic of TC intensification, and its distribution represents a fundamental property of the TC climatology^6^,^7^,^8^.

Several authors have noted that the probability density function (PDF) of global LMI is bimodal. The LMI PDF from International Best-Track Archive for Climate Stewardship for the period 1975–2007 has two local maxima around 40 and 100 kt, and a local minimum at 65 kt (ref. 9). The LMI PDF from a more temporally consistent global data for the period 1982–2009 shows the first maximum at 50 kt and the second one around 120 kt (ref. 10). Considering individual basins, the LMI PDF has its first peak at 50 kt for the North Atlantic, eastern and western North Pacific storms and its second maximum at 110 and 90 kt for eastern and western North Pacific storms^11^. The LMI distribution of Atlantic storms shows no clear secondary maximum, but substantial right skewness^11^. While the precise locations of the LMI PDF peaks vary with the data set and basin, the bimodal feature is quite robust and impossible to overlook. The existence of a second mode means that major storms (sustained winds >96 kt, that is, categories 3–5 TCs in Saffir-Simpson Hurricane Wind Scale) are much less rare than would be expected from the behaviour of the LMI distribution at lower values. The nonmonotonic behaviour of the LMI distribution is in contrast to that of the intensification rate distribution, which is exponential^12^.

Although no complete explanation has been offered in the literature for the bimodality of the LMI distribution, there have been suggestions and indications of what processes might be involved. Uncertainties in the best-track winds is one of them. For example, the widely used Dvorak technique for estimating TC intensity from satellite imagery has low resolution at higher intensities, with only one bin in the category 3 range of the Saffir-Simpson scale. It has been argued that this may result in an artificially low number of category 3 hurricanes (LMI between 96–112 kt) in the Atlantic^13^. A recent study, on the other hand, proposed a parameterization of the ratio of surface exchange coefficients *C*_*k*_/*C*_*d*_, which appears in potential intensity theory^14^,^15^, as a function of wind speed with a local maximum around 115 kt, and speculated that such a maximum would be favourable for rapid intensification (RI) and might explain the bimodal distribution of LMI^5^. In addition to the slope change in *C*_*k*_/*C*_*d*_, observations have suggested an association between RI and eye formation^16^. Organized convective heating results in increased vortex efficiency^17^,^18^,^19^,^20^, which can potentially increase RI probability and cause intense systems. Perhaps most directly relevant, storms reaching the highest intensities in the western North Pacific^21^ and North Atlantic^22^ typically do so after undergoing RI at least once.

Here we extend these results to all basins, and show explicitly that RI explains the bimodality of the LMI distribution. We separate storms into two groups: those that undergo RI during their lifetime (RI storms) and those that do not (non-RI storms). We find that the bimodality of the LMI distribution reflects the mixture of these two unimodal distributions, with the higher intensity peak consisting mostly of storms which have undergone RI at some point. In other words, the LMI distribution is unimodal when RI storms are excluded—RI storms are responsible for the bimodality of the LMI distribution. Various thresholds have been used to define RI^23^,^24^. Here we define RI as an increase of at least 35 kt in the maximum sustained surface wind over a period of 24 h or less. We find this definition of RI to be the most effective in separating the LMI distribution into two unimodal distributions. Sensitivity of the results to other RI thresholds, such as 30 kt, is discussed later.

## Results

### The bimodal LMI distribution

**Figure 1:**
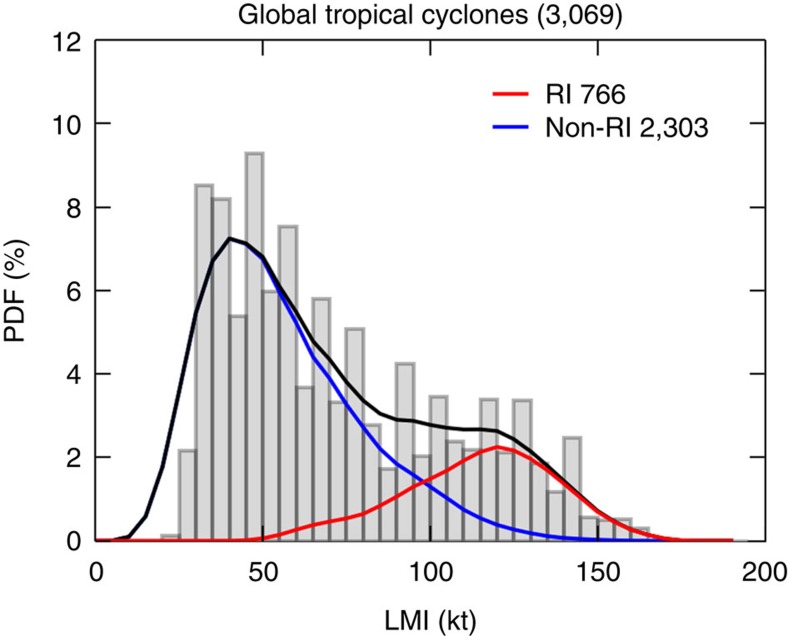
Distributions of global tropical cyclone LMI. PDFs are calculated using 1981–2012 global tropical cyclone LMI. The grey bars show the raw data binned in 5 kt bins. The black, red and blue lines show the smoothed PDF for all storms, storms those undergo rapid intensification during their lifetime (RI storms), and those do not (non-RI storms), respectively. Smoothing is by moving average with window width of 15 kt. Total number of storms is listed in the title, while the numbers of RI and non-RI storms are given in the legend.

**Figure 2:**
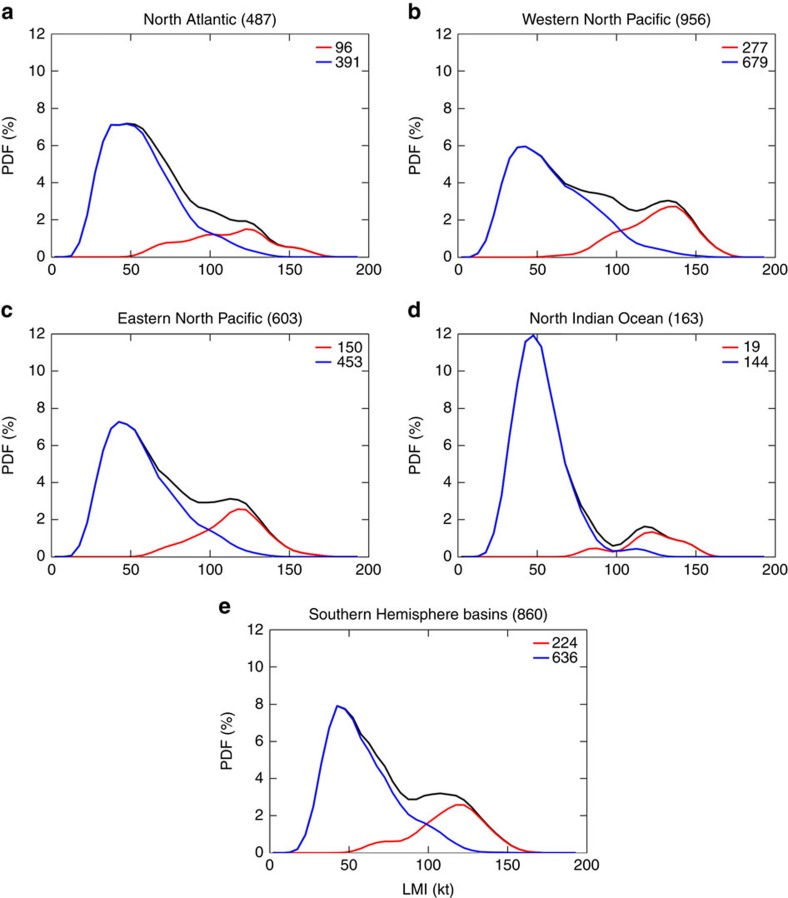
Distributions of regional tropical cyclone LMI. PDFs are calculated using 1981–2012 tropical cyclone for individual basins: (**a**) North Atlantic, (**b**) Western North Pacific, (**c**) Eastern North Pacific, (**d**) North Indian Ocean, and (**e**) Southern Hemisphere basins. The black, red and blue lines show the smoothed PDFs for all, the subset of storms those undergo rapid intensification during their lifetime (RI storms), and those do not (non-RI storms), respectively. The raw data is binned in 5 kt bins and smoothing is by moving average with window width of 15 kt. The number of storms in each basin is given in the title, and the numbers of RI and non-RI storms are given in the legend.

The global distribution of LMI for the period 1981–2012 has a peak at 45 kt and an indication of a secondary maximum around 120 kt (Fig. 1). Basin distributions of LMI are bimodal with local maxima around 45 and 120–135 kt except for the Atlantic where there is only a hint of a second maximum (Fig. 2), similar to what is shown in the literature^11^. The observed LMI distribution shows that category 3 and 4 storms are more common than category 1 and 2 storms. Most types of natural hazards become more rare as they become more extreme, for example, earthquakes^25^ and tornadoes^26^. TCs, as measured by LMI, are unusual in having a range over which frequency increases with intensity. The second peak in the western North Pacific occurs at a higher intensity than in the other basins, consistent with the observation that the stronger storms globally occur more often in that basin^27^.

### Relation to RI

The LMI distribution of the 2,303 non-RI storms in the global record is unimodal and forms the first peak of the complete distribution (blue curve in Fig. 1). About 6% (141) of the non-RI storms are major storms (LMI >96 kt, categories 3–5), and the largest LMI value in this group is 145 kt (category 5). The second peak in the LMI distribution is formed by the 766 RI storms (red curve in Fig. 1), and 79% (603) of them are major storms. The same separation by RI in individual basins yields similar results (Fig. 2). The intensification rate of 35 kt in 24 h (representing the 97th percentile of the intensification rate over that duration) is the optimal RI threshold for explaining the bimodality of the LMI distribution. Using other thresholds, such as 25, 30 or even 40 kt, does not separate the two peaks as clearly, and results a secondary maximum or a hint of it in either the RI or the non-RI LMI distributions (Supplementary Fig. 1). With the 35 kt definition, 80–85% of major storms in the eastern and western North Pacific, North Indian Ocean and southern Hemisphere basins are RI storms, but only 70% of major storms in the Atlantic are RI storms. In the Atlantic, the LMI distribution of RI storms is quite different from that in other basins, being much less peaked (red lines in Fig. 2). The probability that an RI storm in the Atlantic will become a minor hurricane (categories 1–2) is close to the probability that it will become a major hurricane. Still, RI storms are responsible for the rightmost shoulder of the LMI distribution in the Atlantic.

### Relation to storm lifetime

Storm lifetime is another factor related to LMI. The distribution of lifetime itself is unimodal (Supplementary Fig. 2). Overall, LMI is positively correlated with storm lifetime with longer lived storms tending to have higher LMI values. The correlation of storm lifetime with LMI is 0.66 globally. Here the idea that the bimodality in LMI can be explained by variations in lifetime is tested by separating storms into two groups, longer and shorter lived, and examining the LMI distributions for the two groups, analogously to what was done for RI and non-RI storms above. Thresholds of lifetime from 7 to 13 days are tested for this purpose (Supplementary Fig. 3). The classification produces less completely unimodal LMI PDFs than were achieved using RI as the criterion, especially for storms in the North Indian Ocean and southern Hemisphere basins. The best threshold of lifetime is also basin dependent, and noticeable departures from uni-modality are found in even when that best value is used. We, therefore, conclude that while lifetime has an impact on LMI, RI is a better criteria for explaining the LMI bimodality.

## Discussion

The observed relation of RI with the bimodal LMI distribution is consistent with numerical simulation studies in which higher horizontal resolution results in both higher intensification rates and the appearance of the second LMI peak^9^,^28^. Such numerical simulations provide evidence that the observed LMI bimodality and its relationship to RI is not an artifact of the uncertainty associated with intensity estimation^13^,^29^,^30^. Our findings are also in agreement with the hypothesis of ref. 5 that RI is responsible for the bimodal distribution of LMI, although we cannot comment on the link to the ratio of exchange coefficients. On the other hand, a recent study showed that RI has no apparent signature in the overall intensification rate statistics^12^. The intensification rate distributions of RI and non-RI storms do differ, even when the RI events are excluded, but no distinguishing features result when all storms are considered (Supplementary Fig. 4). A key difference between the distribution of intensification rates and that of LMI is that the LMI distribution accounts for the intensification behaviour over the storm lifetime, and thus is a measure of the joint (across different times) intensification rate statistics.

Another measure of the TC intensity climatology is LMI normalized by local potential intensity. The distribution of normalized LMI for storms whose peak intensities are not limited by declining potential intensity has a linear cumulative distribution with two slopes^6^, corresponding approximately to tropical storms (>34 kt) and hurricanes (>64 kt). This separation of the normalized LMI distribution appears to be distinct from the one here based on RI. The subset of storms not limited by declining potential intensity is a relatively small fraction of all storms, and the normalized LMI distribution over all storms (which we consider here) is not uniform (not shown; for example, Fig. 13 in ref. 6).

The basic physical mechanisms of RI are still not completely understood, and whether they are distinct from those of lower intensification rates remain an open question. We do not attempt to answer this question. The message here is that RI is relevant not only to short-term weather forecasting, but also to the relationship between TCs and climate. The most intense storms are those that undergo RI, and the storms that undergo RI are responsible for the observed bimodality of the LMI distribution. This finding suggests that a complete understanding of the most intense storms in either the current climate or future (or past) climates may need to include some understanding of RI. Our results also suggest that numerical models that do not simulate RI are likely to be incomplete in their representation of the LMI distribution and in the frequency of major storms. Therefore, an important research question is to what extent simulations and projections of the frequency of major storm occurrence can be accurate without either resolving RI or accounting for its absence.

## Methods

### Data

Best-track data from the National Hurricane Center (NHC)^29^,^31^ and the Joint Typhoon Warming Centers (JTWC)^32^ from 1981 to 2012 are used in this study. Best-track data include 1-min maximum sustained wind, minimum sea level pressure and location every 6 h.

### Calculations

The LMI here is defined using maximum wind speed. Maximum wind speed itself is not an observed quantity, but rather estimated from *in situ* observations, remotely sensed estimates of winds or via satellite-based techniques^29^. The distribution of LMI is calculated globally, as well as for individual basins, following the definitions of the NHC and JTWC, that is, North Atlantic and eastern North Pacific (from NHC), and western North Pacific, North Indian Ocean and southern Hemisphere basins (from JTWC). Correlations between global LMI and storm lifetime are calculated using Spearman's rank correlation coefficient.

### Terminology

We refer to storms with LMI >96 kt (categories 3–5 in Saffir-Simpson Hurricane Wind Scale) as major storms.

## Additional information

**How to cite this article:** Lee, C-Y. *et al.* Rapid intensification and the bimodal distribution of tropical cyclone intensity. *Nat. Commun.* 7:10625 doi: 10.1038/ncomms10625 (2016).

## Supplementary Material

Supplementary Information Supplementary Figures 1-4
