# Supplementary material for: Rapid intensification and the bimodal distribution of tropical cyclone intensity
Source: Nat Commun. 2016 Feb 3;7:10625. doi: 10.1038/ncomms10625 (PMC4742962; doi:10.1038/ncomms10625)
Supplement: Supplementary Information — Supplementary Figures 1-4 [file ncomms10625-s1.pdf]

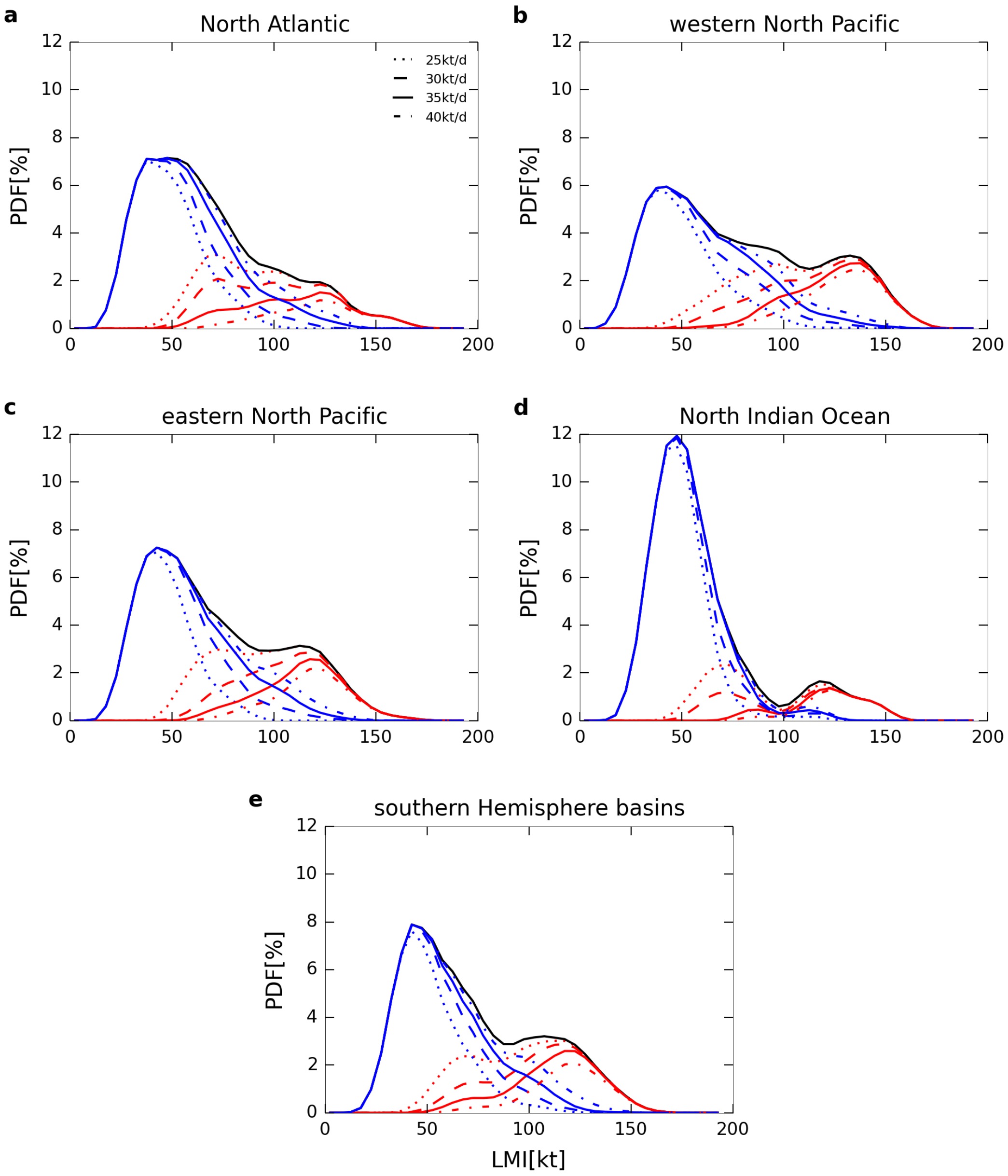

Supplementary Figure 1. Probability density functions (PDFs) of tropical cyclone lifetime maximum intensity (LMI) for individual basins. The black, red and blue lines show the smoothed PDFs for all, RI storms, and non-RI storms, respectively. Smoothing is by moving average with window width of 15 kt. Line style indicates the RI-definition used, as labeled in the legend.

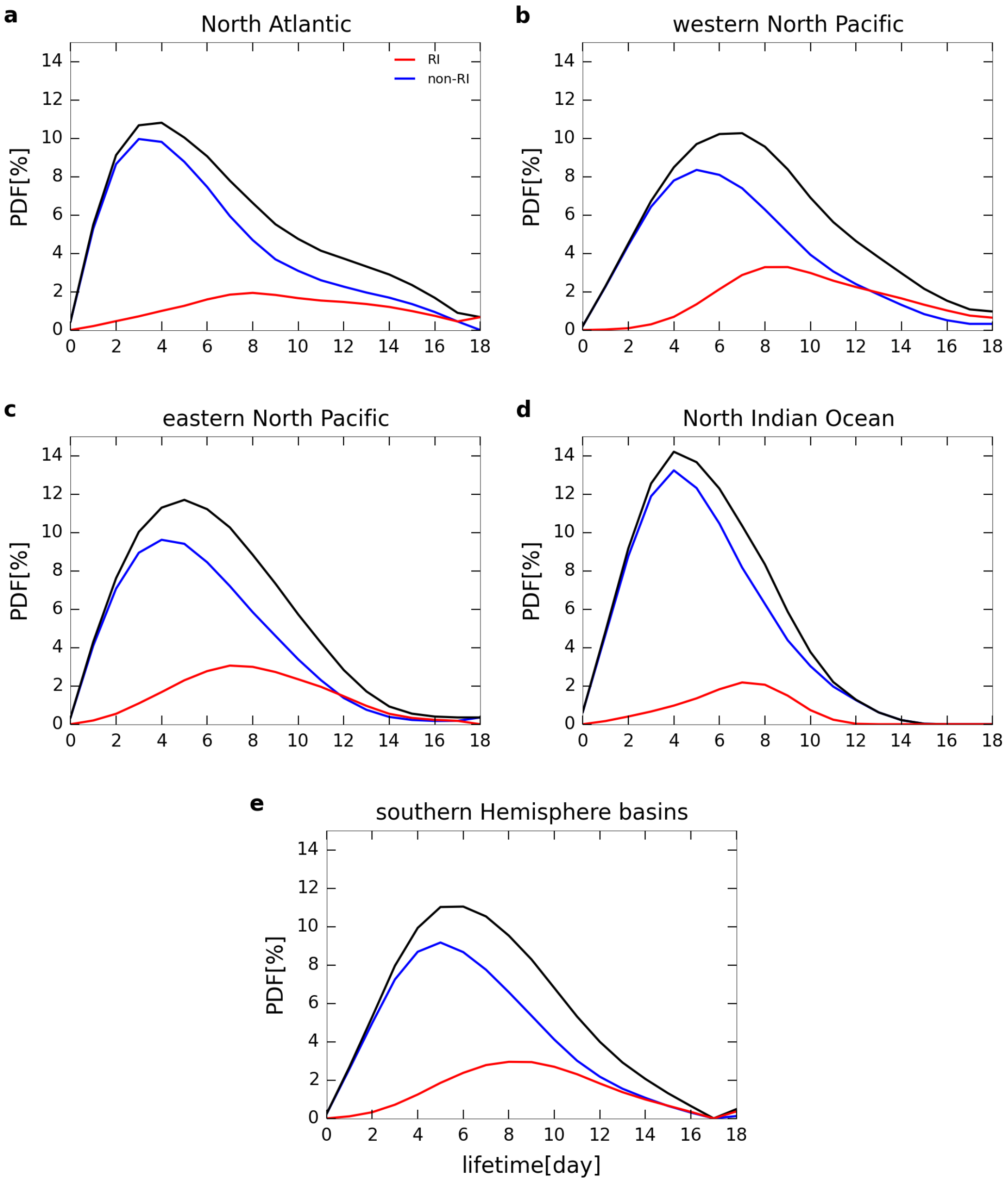

Supplementary Figure 2. PDFs of storm lifetime for individual basin. The black, red, and blue lines show the smoothed PDFs for all, RI storms, and non-RI storms, respectively. Smoothing is by moving average with window width of 3 days. RI is defined as an increase of at least 35 kt in the maximum sustained surface wind over a period of 24 hours or less.

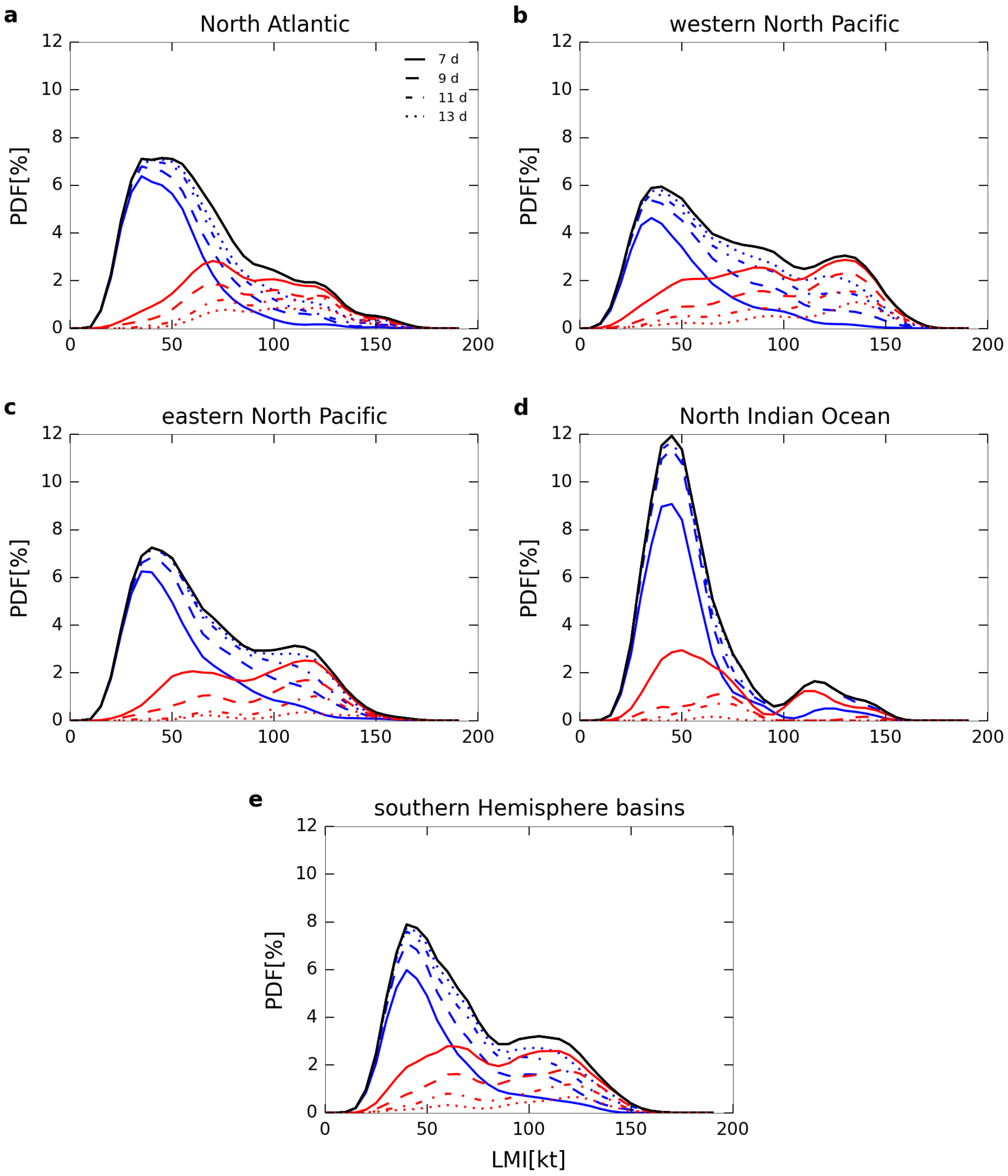

Supplementary Figure 3. PDFs of LMI for individual basin. The black, red, and blue lines show the smoothed PDFs for all, storms with lifetime longer, and less than the given threshold, respectively. Smoothing is by moving average with window width of 15 kt. Line style indicates the lifetime threshold used, as labeled in the legend.

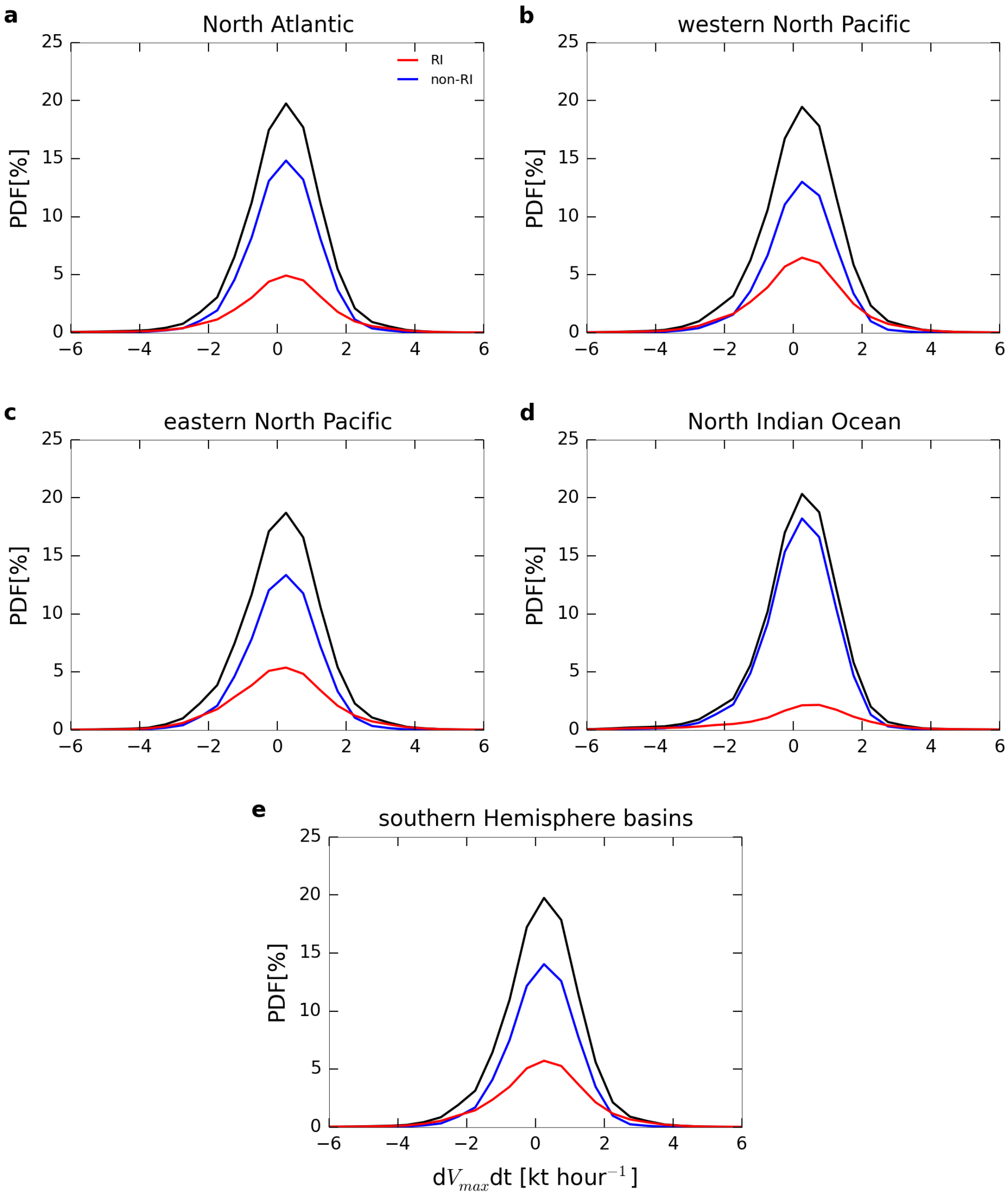

Supplementary Figure 4. PDFs of intensification rate for individual basin. The black, red, and blue lines show the smoothed PDFs for all, RI storms, and non-RI storms, respectively. Smoothing is by moving average with window width of 1.5 kt hour^{-1}. RI is defined as an increase of at least 35 kt in the maximum sustained surface wind over a period of 24 hours or less.
